# Large-Scale Spatio-Temporal Patterns of Mediterranean Cephalopod Diversity

**DOI:** 10.1371/journal.pone.0146469

**Published:** 2016-01-13

**Authors:** Stefanie Keller, Valerio Bartolino, Manuel Hidalgo, Isabella Bitetto, Loredana Casciaro, Danila Cuccu, Antonio Esteban, Cristina Garcia, Germana Garofalo, Marios Josephides, Angelique Jadaud, Evgenia Lefkaditou, Porzia Maiorano, Chiara Manfredi, Bojan Marceta, Enric Massutí, Reno Micallef, Panagiota Peristeraki, Giulio Relini, Paolo Sartor, Maria Teresa Spedicato, George Tserpes, Antoni Quetglas

**Affiliations:** 1 Instituto Español de Oceanografía, Centro Oceanográfico de Baleares, Muelle de Poniente, s/n, Apdo. 291, 07015 Palma de Mallorca, Spain; 2 SLU Swedish University of Agricultural Sciences, Department of Aquatic Resources, Lysekil, Sweden; 3 COISPA-Tecnologia & Ricerca, Stazione Sperimentale per lo Studio delle Risorse del Mare, Bari, Italy; 4 Dipartemento di Scienze della Vita e dell’Ambiente, Università di Cagliari, Cagliari, Italy IEO; 5 Centro Oceanográfico de Murcia, San Pedro del Pinatar, Murcia, Spain; 6 IEO, Centro Oceanográfico de Malaga, Fuengirola, Málaga, Spain; 7 IAMC–Coastal Marine Environment Insitute–CNR, Mazara del Vallo (TP), Italy; 8 DFMR–Department of Fisheries and Marine Research, Ministry of Agriculture, Rural Development and Environment, Nicosia, Cyprus; 9 Ifremer, Institut Français de Recherche pour l'Exploitation de la mer, UMR 212 Ecosystèmes Marins Exploités (EME), Sète, France; 10 HCMR, Hellenic Centre of Marine Research, Athens, Greece; 11 University of Bari Aldo Moro—Department of Biology, Bari, Italy; 12 Laboratorio Biologia Marina e Pesca, Università di Bologna, Fano (PS), Italy; 13 Fishery Research Institute of Slovenia, Ljubljana-Smartno, Slovenia; 14 Ministry for Sustainable Development, Department of Fisheries and Aquaculture, Marsa, Malta; 15 HCMR, Hellenic Centre of Marine Research, Heraklion, Crete, Greece; 16 SIBM, Società Italiana di Biologia Marina, Genova and DISTAV, Università di Genova, Genova, Italy; 17 CIBM–Centro Interuniversitario di Biologia Marina ed Ecologia Applicata, Livorno, Italy; Tuscia University, ITALY

## Abstract

Species diversity is widely recognized as an important trait of ecosystems’ functioning and resilience. Understanding the causes of diversity patterns and their interaction with the environmental conditions is essential in order to effectively assess and preserve existing diversity. While diversity patterns of most recurrent groups such as fish are commonly studied, other important taxa such as cephalopods have received less attention. In this work we present spatio-temporal trends of cephalopod diversity across the entire Mediterranean Sea during the last 19 years, analysing data from the annual bottom trawl survey MEDITS conducted by 5 different Mediterranean countries using standardized gears and sampling protocols. The influence of local and regional environmental variability in different Mediterranean regions is analysed applying generalized additive models, using species richness and the Shannon Wiener index as diversity descriptors. While the western basin showed a high diversity, our analyses do not support a steady eastward decrease of diversity as proposed in some previous studies. Instead, high Shannon diversity was also found in the Adriatic and Aegean Seas, and high species richness in the eastern Ionian Sea. Overall diversity did not show any consistent trend over the last two decades. Except in the Adriatic Sea, diversity showed a hump-shaped trend with depth in all regions, being highest between 200–400 m depth. Our results indicate that high Chlorophyll *a* concentrations and warmer temperatures seem to enhance species diversity, and the influence of these parameters is stronger for richness than for Shannon diversity.

## Introduction

Species diversity has strong implications on the functioning and conservation state of ecosystems. Its preservation should therefore be a priority in conservation management, and indeed many protected areas are established based on the diversity hotspots they sustain. Understanding the causes of underlying diversity patterns and their interaction with the environmental conditions are of paramount importance for its conservation. Species distributions are not always explicable by their physiological constraints, and the mechanisms structuring diversity patterns are often unknown [1]. On a global scale, different drivers have been proposed up to now, including factors like ecosystem productivity, climate and habitat heterogeneity, as well as historical causes or geographical barriers [2]. The latitudinal gradient (poleward decrease) of diversity is the most striking pattern and has been investigated for different marine taxa (e.g. [3]; [4], [5]). In the marine environment, sea temperature and productivity are commonly reported as the main drivers of geographical distribution patterns of diversity [6–9].

The Mediterranean Sea is a semi-enclosed basin characterized by pronounced longitudinal gradients in temperature and productivity. Accordingly, an eastward decreasing trend in fish species richness has been reported by some authors [1,10,11]. This trend has often been explained by the large-scale eastward decline in primary production which is observed in the Mediterranean [6], arguing that areas of high food availability serve as feeding and reproduction sites for many species [11]. Temperature regime is another possible determinant of diversity, affecting the competitiveness of animals via their different temperature tolerances and mobility [9]. In addition to food supply and temperature, the inflow of Atlantic water and human influences have been identified as local-scale factors structuring patterns of spatial diversity in the Mediterranean [12–14].

While there have been some studies on Mediterranean fish diversity [12,15,16], no large scale analysis of Mediterranean cephalopod diversity exists. This contrasts with the fact that they play a key role in marine ecosystems, as they are important prey species as well as voracious predators [17]. Therefore, fluctuations in their community composition and abundance are likely to have profound consequences for the food webs and ecosystems. Furthermore, cephalopods are important living resources for most Mediterranean countries, sustaining some economically important fisheries in various areas [18]. Being semelparous species with a short generation time of normally one to two years, they can respond rapidly to changes in environmental conditions [19]. Temporal changes in their abundance and diversity can therefore supply important information about ecosystem alterations. Despite their importance, the only existing publications looking at their large-scale diversity patterns are species inventories comparing species richness between the Adriatic Sea, the western and the eastern Mediterranean basin [20,21]. Mangold and Boletzky [20] conclude a general eastward decrease in species numbers and found the Adriatic to contain the lowest species richness, but the more recent study of Bello [21] noted fewer differences between the areas. This is characteristic for species inventories, which, although historically very precious, suffer from biases resulting from different sampling intensities [22]. More analytical cephalopod diversity studies are restricted to local scales, both in the western [23–25] and the eastern [26–28] basins. At such local scales, depth was found to be the main diversity driver.

Understanding and monitoring species diversity is a crucial issue in confined areas with substantial human influences such as the Mediterranean Sea [14]. Climate change [29], resource exploitation [30,31], pollution [32] and other anthropogenic influences like habitat loss and the introduction of alien species [13] steadily impact the local diversity, with unknown implications for the concerned area. Despite this, few diversity studies covering the whole longitudinal gradient of the Mediterranean Sea exist until now, and long-time data series based on standardized protocols are rare (but see [12,15,16]). One dataset of this kind results from the international Mediterranean bottom trawl survey MEDITS, which is performed every spring since 1994 [33]. Due to its regular realization, large-scale coverage, the length of the time series and the standardization of methodology, this dataset is one of the most valuable data sources to investigate large scale patterns of demersal species abundance and diversity in the Mediterranean Sea.

Using the MEDITS dataset we investigate the relationship between demersal cephalopod diversity and environmental characteristics. The aim of this work is two-fold. First, we explore cephalopod spatio-temporal diversity patterns in the Mediterranean, using the entire time series of MEDITS cruises currently available (19 years). Secondly, using a shorter time series, we analyse the influence of putative environmental covariates on the spatial and temporal variation of cephalopod diversity.

## Material and Methods

### Data sources

**Fig 1:**
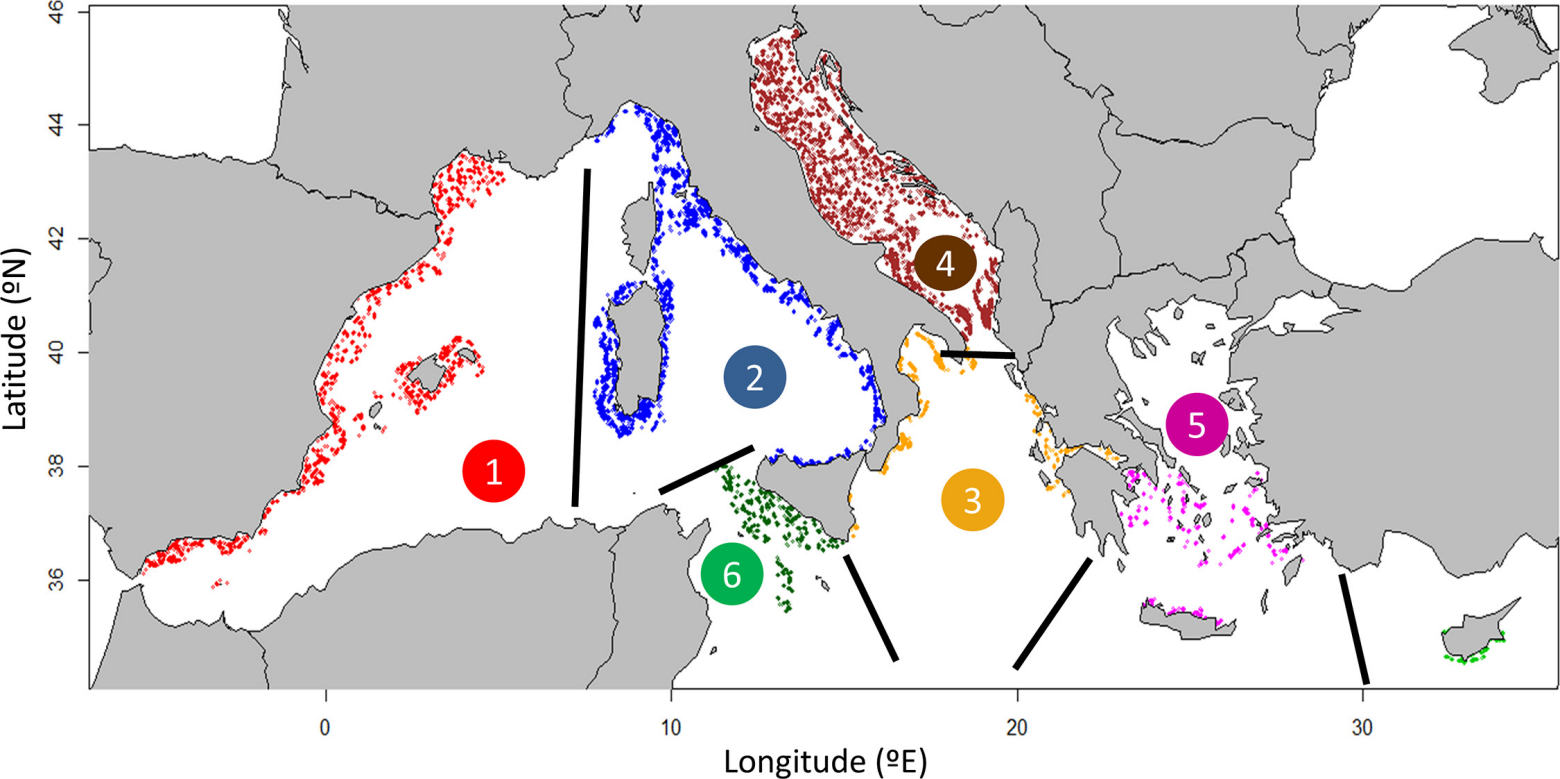
Map of the Mediterranean Sea showing the MEDITS stations sampled during 1994–2012. Plotted are 20463 hauls and colours correspond to the following bioregions: 1) Iberian-Lions, 2) Tyrrhenian Sea, 3) Adriatic Sea, 4) Ionian Sea, 5) Aegean Sea and 6) Strait of Sicily. Separation of bioregions is marked with thick black lines.

The international Mediterranean bottom trawl survey MEDITS (www.sibm.it/SITO%20MEDITS/) is performed by all riparian EU countries, and also by Montenegro and Albania. The sampling was performed under repeated international standardized protocol (for details see [33]). The research vessels had full permission from national (Fisheries General Secretariat) and international authorities (General Fisheries Commission for the Mediterranean) to sample in territorial and Mediterranean community waters. No approval by an ethics committee was required as common exploited species were targeted and trawling did not affect endangered or protected species or marine protected areas. Most of the authors participate consistently in the surveys of the MEDITS programme. Since 1994, the survey is conducted every year in spring / early summer (May-August) and covers depths from 10 m down to 800 m. The MEDITS area is divided into different geographical sub-areas (GSA’s; S1 Fig), established by the General Fisheries Commission for the Mediterranean (www.gfcm.org). The sampling procedure is standardised, with a common sampling protocol and strategy in place and the same gear employed throughout the whole study zone [33]. The area of available data ranges from 5.21° W to 27.75° E, and from 35.22° to 45.65° N across the national waters of 10 countries (Fig 1). A stratified random sampling design is used for this survey, with bathymetric strata comprising 10–50, 51–100, 101–200, 201–500 and 501–800 m. The standardized gear used is a GOC 73 trawl with a cod-end mesh size of 20 mm and a vertical and horizontal opening of the net of about 2 m and 18 m, respectively [33]. The net opening is measured by an attached underwater Scanmar system, which allows calculating the swept area. Trawling is conducted at daylight, with a towing speed of 2–3 knots and hauls duration of 30 and 60 minutes over shelf and slope grounds respectively. Haul catches are sorted to species level whenever possible. Abundance data for each species are standardized to number of individuals per km^2^ using the swept area method.

### Diversity measures and modelling approach

Two different diversity measures, widely used in fishery studies, were selected for this study in order to capture the main axes of biodiversity variability: the Shannon-Wiener Diversity (H’) and the species richness index (S). While S is the total number of species, H’ takes into account the number of species as well as their contribution within a sample (evenness). Given the same number of species, samples with more even abundances (i.e., less dominant species) have a higher H’. Garofalo et al. [34] evaluated correlations among different indices of diversity performing Spearman rank tests and showed that the Shannon Wiener index evidenced the strongest positive correlation with the other indices they analyzed. In this study, S and H’ were chosen as they give complementary information. Other diversity indices (e.g. Simpsons index or rarity) have not been used to avoid redundancy [15]. Both indices were calculated based on species abundance data.

Prior to final analyses, exploratory analyses of spatio-temporal trends were performed on all data from 1994–2012. Some areas only sampled in recent or in very few years where excluded from these analyses (GSA 2, GSA 15, GSA 25). Greece (GSA 20, 22, 23) did not conduct the MEDITS surveys in 2002, 2007 and in 2009–2012, so the overall Mediterranean diversity in recent years is not completely comparable to former years. Also, in some years and areas around Greece, not all cephalopod species were recorded; therefore the respective surveys were excluded as well. In 1997, GSA 23 had to be excluded for this reason, and therefore few stations were sampled in the Southern Aegean Sea in that year. In the same area, all samples between 1998–2001 had to be excluded. Despite these deficiencies, the Greece data were included as they represent the easternmost data points of the time series. The final dataset included 15 GSAs and comprised 18214 hauls (S1 Fig).

For analysis purpose, the Mediterranean was divided into 6 biogeographical zones modified from Gaertner el al. [15] (Fig 1). These bioregions represent ecologically meaningful units rather than artificial management units as do the GSAs. As diversity index values are scale-dependent, different spatial scales (haul, GSA and bioregion) are considered. We computed the diversity at haul scale (α-diversity) and the total diversity over all hauls both per GSA (γ-diversity) and per bioregion. While the modelling and the results presented here are based on α-diversity and rarified species richness per bioregion, the results by GSA (γ-diversity) are given in the supplementary material (S2 Fig). As species richness per spatial unit depends on the sampling effort, the number of total species at each bioregion / GSA was rarefied to 35 / 20 hauls respectively [16]. Sample-based rarefaction is a method to calculate species richness for a given number of individual samples by randomly re-sampling the pool of N samples many times; then the average number of species found in each sample can be calculated [35]. In our case, the use of 35 /20 hauls was a trade-off between excluding areas with less hauls and having a representative sample size for all regions (trials were also made with different numbers of hauls but gave the same pattern). Furthermore, species accumulation curves, which show the accumulated number of species for each additional sample taken, were calculated; these curves reach an asymptote once further sampling does not yield any new species.

To analyse synchrony of trends between bioregions, Pearson correlation coefficients were calculated between the time series of the six bioregions. Correlation coefficients, diversity indices, rarefaction and species accumulation curves were calculated in R (version 2.15.1; http://www.r-project.org/) using the vegan package (2.0–10).

When modeling putative drivers of H’ and S, only surveys from 2003–2008 were included, as in these years most areas were sampled consistently. To account for the skewed sampling in shallower areas where very few hauls deeper than 700 m were available, only hauls down to this depth were considered. The final data used came from 15 GSAs and comprised 6275 hauls. As we do not have prior expectations on the relationships between diversity and its drivers and cannot exclude the occurrence of non-linearity, Generalized Additive Models (GAMs) were used. A two-dimensional smoother was used combining latitude and longitude to account for the spatial effect. The variable ‘year’ was considered as factor, while for ‘depth’, a general effect was tested versus various regional effects (where depth was modelled separately for each bioregion) as we expected spatial differences. Mean seasonal chlorophyll-a concentration (Chla; mg·m^-3^) and Sea Surface Temperature (SST; °C) were averaged across GSAs; whereas SST was normally distributed, Chla values were log-transformed to obtain normalized distributions. Chla concentration was used as a proxy for food availability and was modelled using seasonal means of the winter (December-February) and spring (March-May) preceding the survey, in order to account for the time required for energy transfer between trophic levels. The same two seasons were used to calculate mean seasonal SST, as temperature might influence ecological and metabolic processes differently at different stages of the animal’s life history. These covariates and periods of year have been selected as good descriptors of the main oceanographic processes determining spring productivity in the Mediterranean [36,37]. Chla and SST data were obtained from satellite-derived products available on the Giovanni NASA webpage (http://gdata1.sci.gsfc.nasa.gov). The model formulation was as follows:

Species diversity:H’∼Year+s(Lat,Long)+Depth*Bioregion+s(Chla)+s(SST)

Species richness:S∼Year+s(Lat,Long)+Depth*Bioregion+s(Chla)+s(SST)

Best model selection was based on the minimization of both the Generalized Cross-Validation (GCV) and the Akaike Information Criterion (AIC). All GAM analyses were carried out with R using the mgcv library (1.7–29). For all GAMS, residual plots were checked and confirmed the assumptions of variance homogeneity and normal distribution. Using decorrelation plots, we assured the existence of spatial autocorrelation of the diversity index values, a prerequisite to build spatial models. Finally, residuals were checked for the absence of spatial correlation with directional variograms and spatial plotting of residuals, assuring us that the model covariates account for the spatial variation of diversity.

## Results

**Fig 2:**
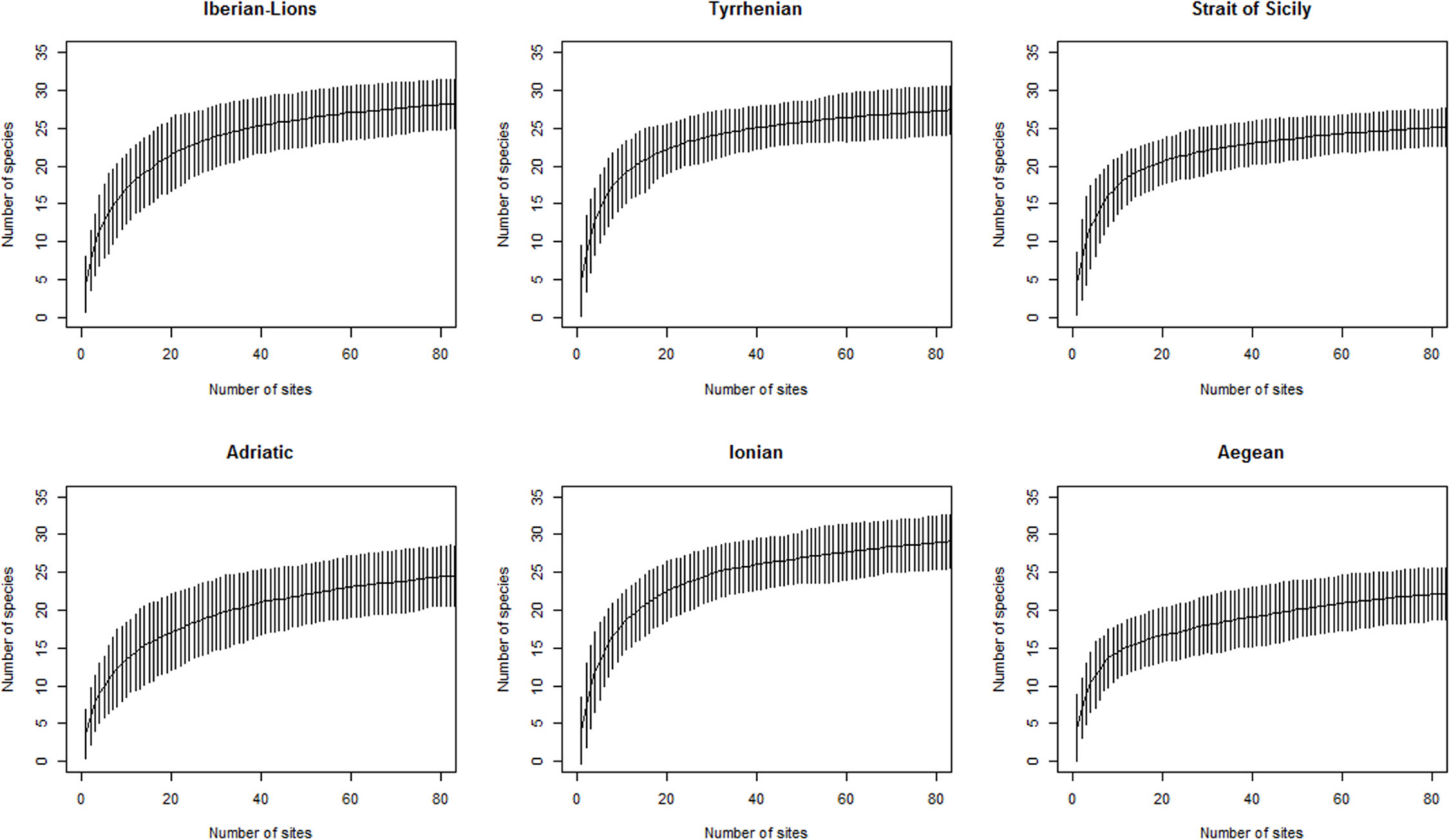
Species accumulation curves of MEDITS sampling at different Mediterranean bioregions.

During all cruises, a total of 58 species or taxa of cephalopods were found (see S1 Table), of which 47 were determined to species level. In spite of the large number of sampling stations analysed, the species accumulation curves (Fig 2) have not completely leveled off in most bioregions, which means that additional sampling is required to catch all the regional cephalopod diversity.

### Exploratory analysis

**Fig 3:**
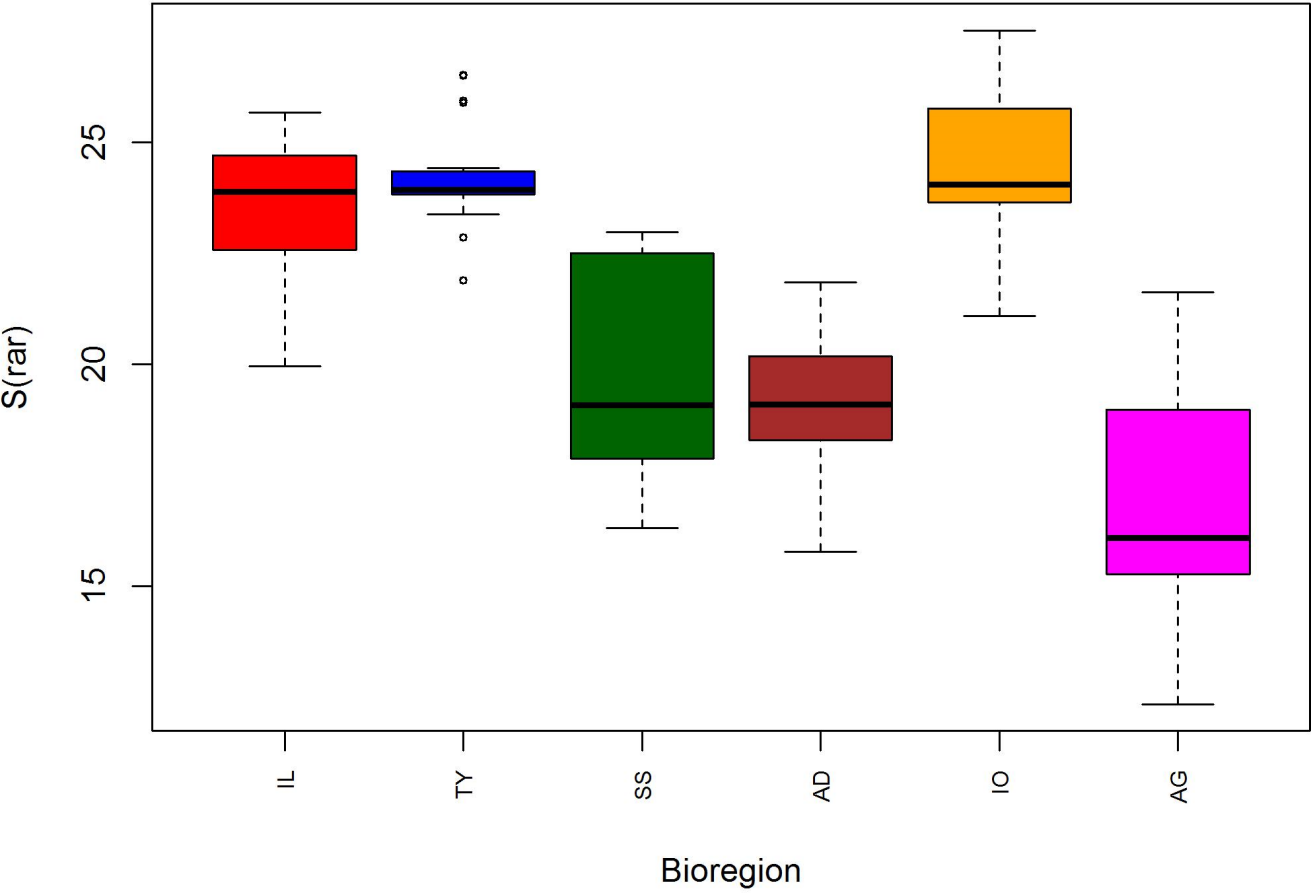
Boxplot of total species richness (S_rar_) at different Mediterranean bioregions: Iberian-Lions (IL), Tyrrhenian Sea (TY), Strait of Sicily (SS), Adriatic Sea (AD), Ionian Sea (IO) and Aegean Sea (AG). Samples were included from 1994–2012 and rarefied to 35 hauls.

The 47 cephalopods determined at species level were included in this analysis. When joining all data from 1994–2012 and rarefying species number to 35 hauls, the highest and lowest median species richness recorded were 24 and 16 species in the Ionian and Aegean Sea respectively (Fig 3). While the Iberian-Lions (S = 23.88) region and the Tyrrhenian Sea (S = 23.93) also showed high species richness, the Adriatic Sea (S = 19.10) and the Strait of Sicily (S = 19.75) yielded comparatively fewer species.

**Fig 4:**
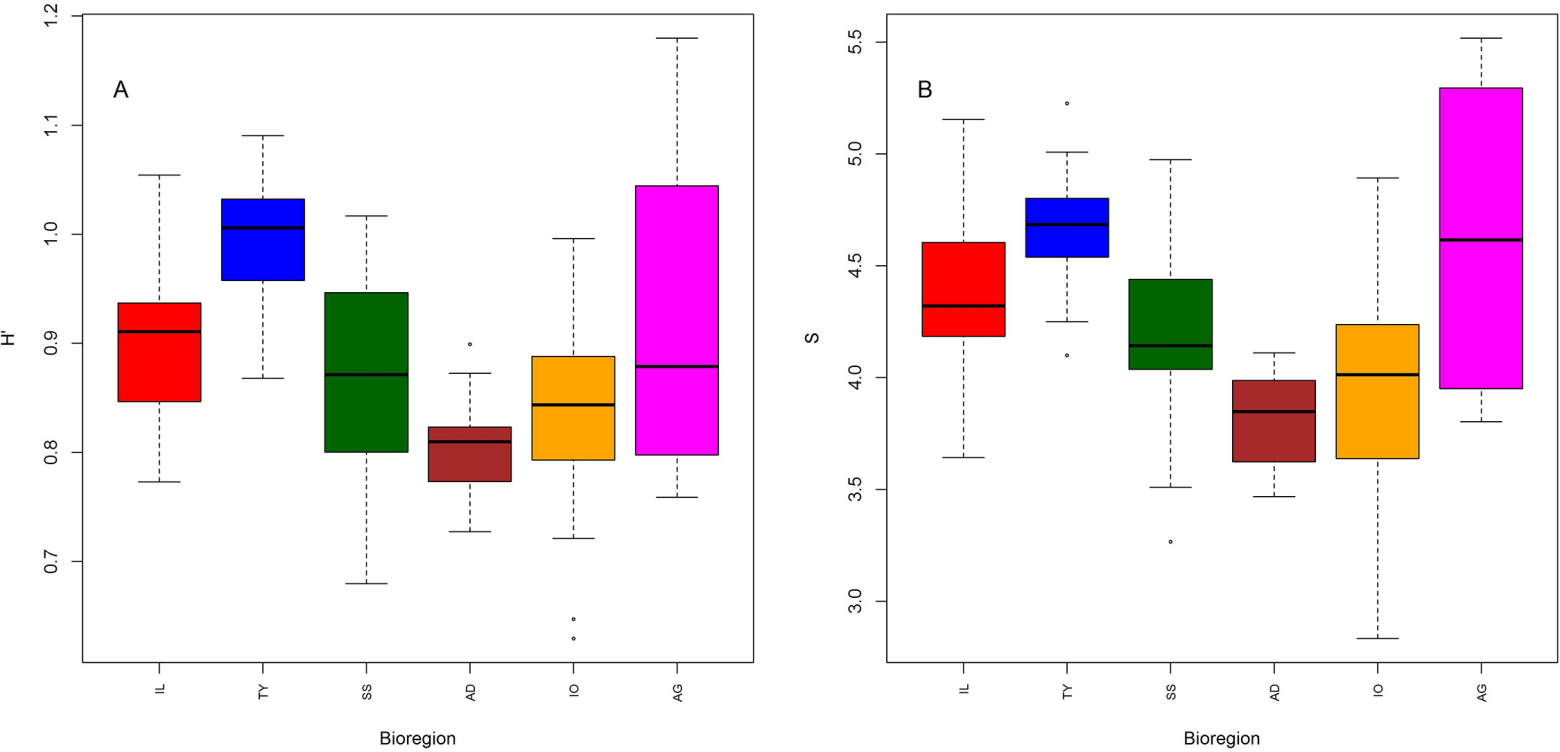
Shannon diversity (A) and Species richness (B) per haul from MEDITS sampling (1994–2012), calculated for the following bioregions: Iberian-Lions (IL), Tyrrhenian Sea (TY), Strait of Sicily (SS), Adriatic Sea (AD), Ionian Sea (IO) and Southern Aegean Sea (AG).

Except in the Aegean Sea, Shannon diversity (H’) and species richness (S) per haul (α-diversity) showed the same bioregional trend, being highest in the Tyrrhenian Sea and lowest in the Adriatic Sea (Fig 4). In the Aegean Sea, H’ and S showed contrasting values, with S being very high but H’ comparatively low. Species richness within one GSA (Gamma diversity) shows a quite variable pattern, from a median of 14 species (GSA 7) to 22 species (GSA 1 and 9) (S2 Fig). The Alboran Sea, the Catalan Sea and the Balearic Sea are characterized by a high number of cephalopod species, and similar high richness values can be found in the Tyrrhenian (GSA 9, 10) and the Ionian Sea (GSA 19, 20). The waters around the Strait of Sicily (GSA 16) and Sardinia (GSA 11) are slightly less diverse, and the lowest richness can be found in the Gulf of Lions (GSA 7), the Sea around Corsica (GSA 8) and the Adriatic and Southern Aegean Sea.

**Fig 5:**
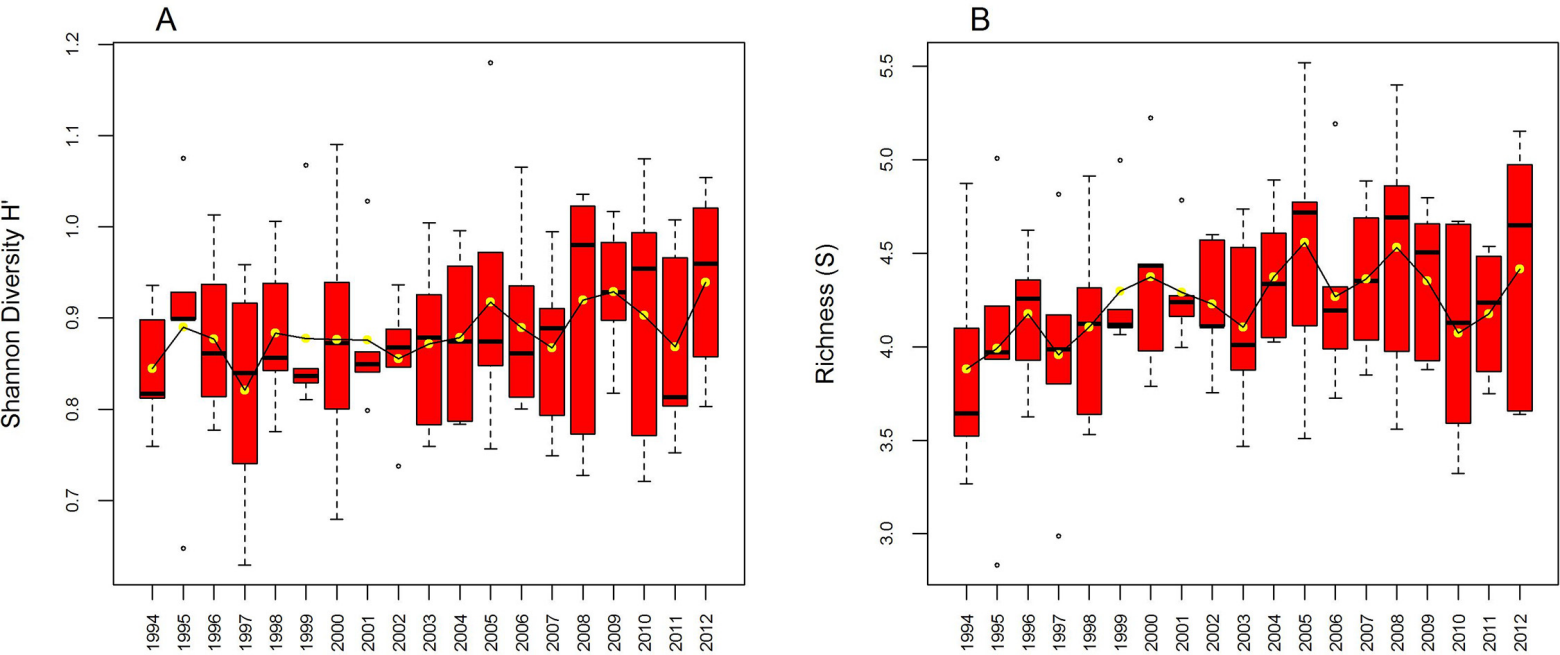
Boxplots of Shannon diversity (A) and Species richness (B) per haul, obtained using all MEDITS samples taken during 1994–2012.

**Fig 6:**
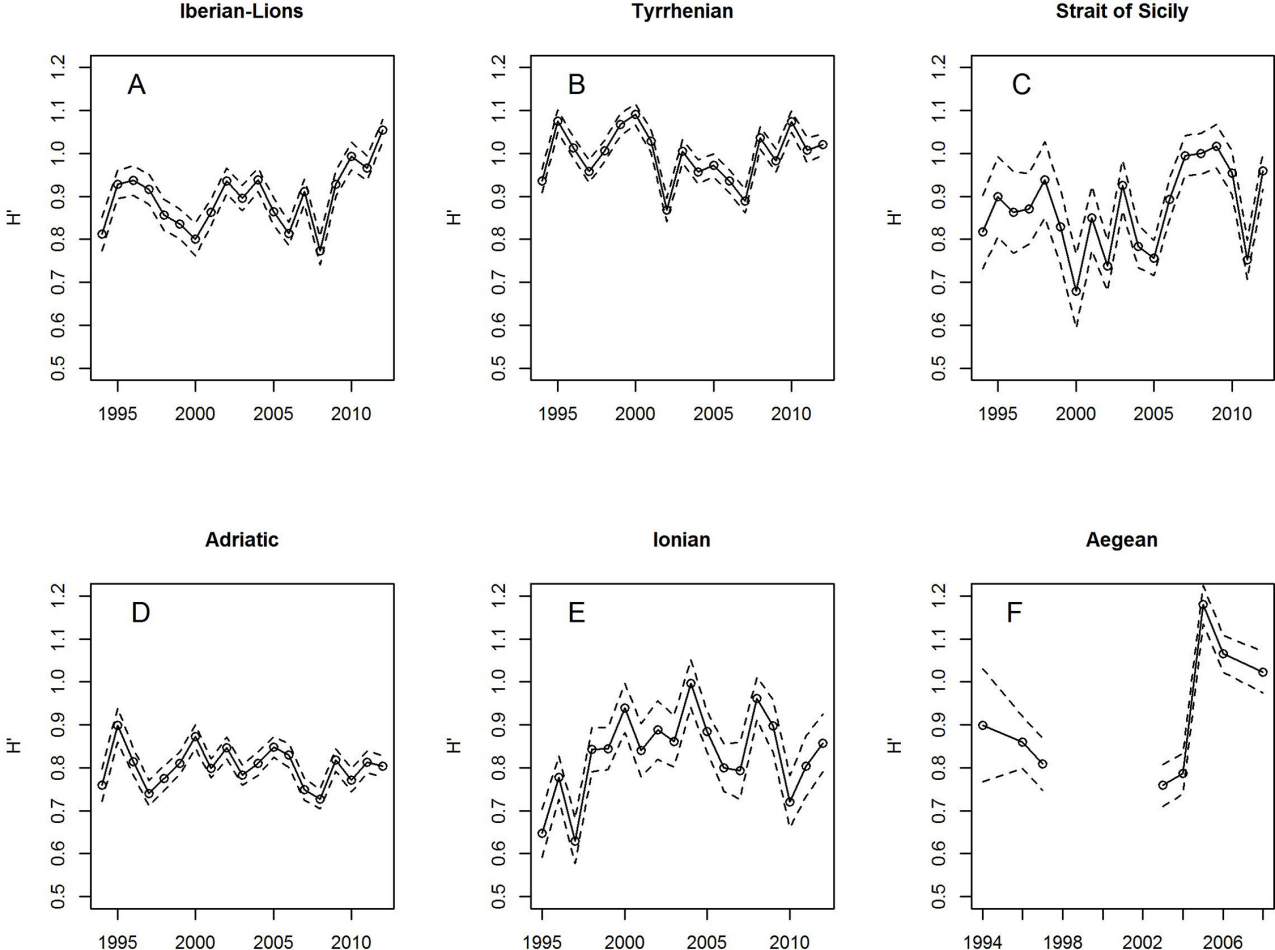
Temporal patterns of the Shannon diversity H’ at six different Mediterranean bioregions using the MEDITS sampling carried out during 1994–2012.

The mean diversity and species richness over the whole Mediterranean did not show any temporal trend during the study period (1994–2012), with mean H’ ranging between 0.81 and 0.94 and mean S between 3.8 to 4.5 species per haul (Fig 5). The lack of consistent temporal trends of H’ and S also applied to regional level (Fig 6; as H’ and S displayed a very similar pattern, only H’ is shown). The absence of significant cross-correlations among temporal variation of H’ indices of different areas (with the exception of the Adriatic with the Strait of Sicily time series, p = 0.0238) evidences the general absence of synchronic behavior.

### Environmental drivers

The best models for H’ and S were obtained including the factor year, a two-dimensional smoother for longitude and latitude, a depth-region interaction term, and smoothers for Chla and SST (S2 Table). The depth-region interaction term indicates that the bathymetric pattern of diversity varies with the bioregion considered. The best fits for both models (H’ and S) were obtained using the Chla of the winter preceding the sampling season. Regarding the SST, the best models for H’ included the SST values of previous winters, while the richness model performed better using the spring SSTs. The deviance explained was much higher for the S model (35.9%) than the H’ model (18.5%).

**Fig 7:**
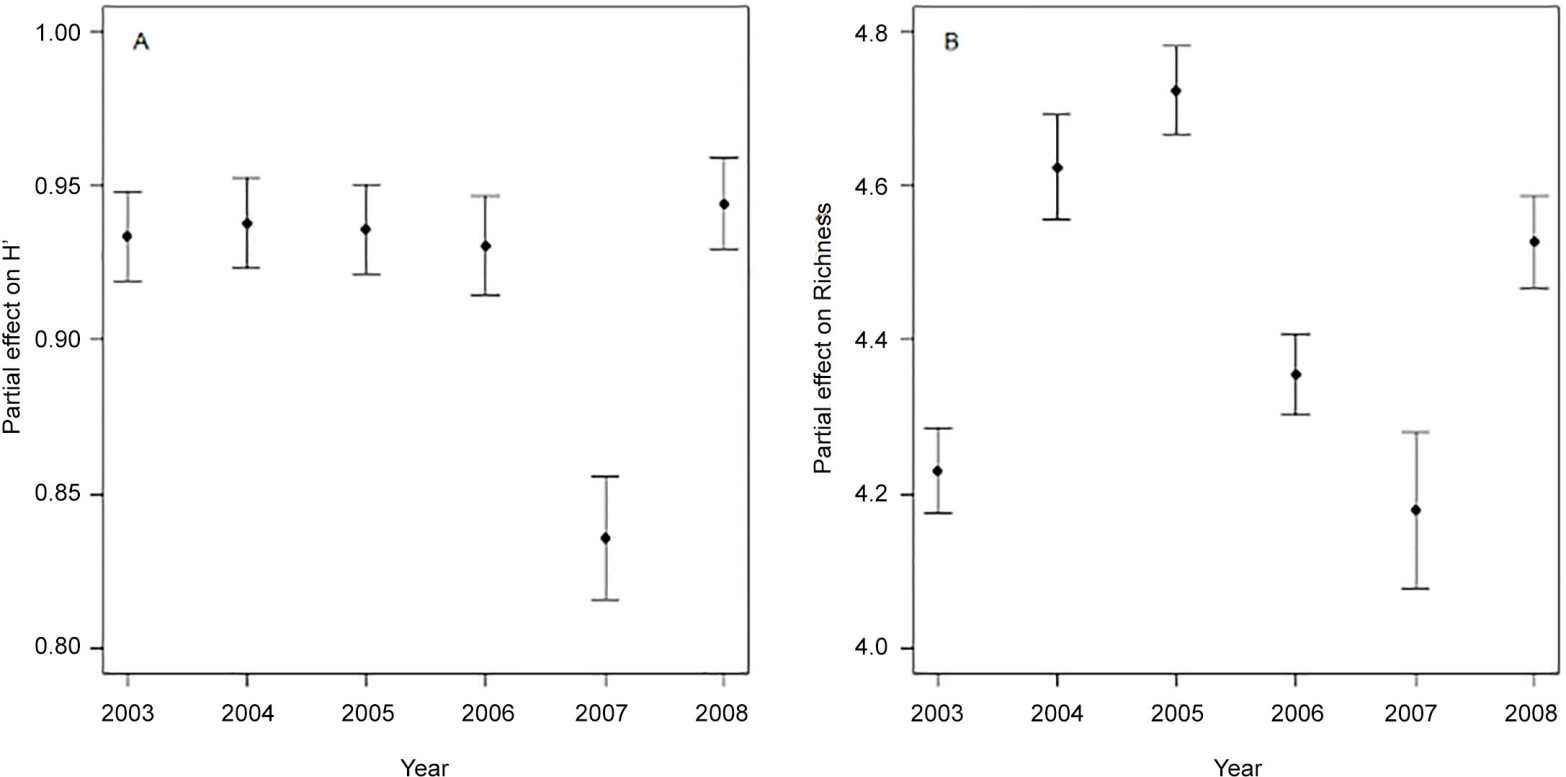
GAM outputs for partial effects for factor “year” (mean ± S.E.) on Shannon diversity (A) and species richness (B) of Mediterranean cephalopods collected during MEDITS sampling (2003–2008).

**Fig 8:**
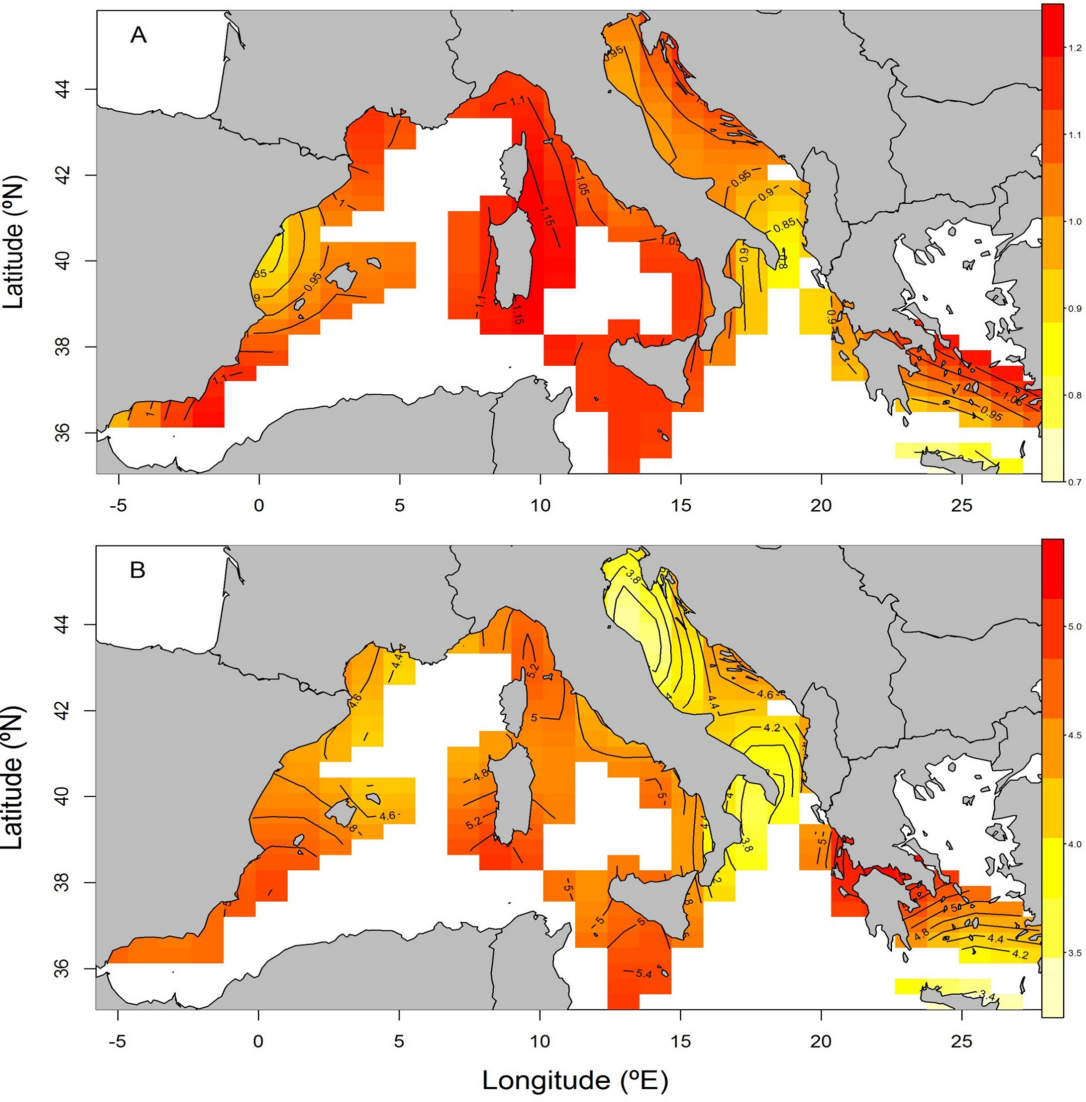
Spatial pattern of Shannon diversity H’ (A) and species richness S (B) across the Mediterranean Sea as predicted by the GAM model.

Modeling H’, the partial effect of factor year was quite stable over the time series analyzed, with the only exception of 2007, whereas it varied without any clear trend for S (Fig 7). The spatial analyses (two-dimensional smoother for longitude and latitude) revealed slightly contrasting patterns between diversity and species richness (Fig 8). Overall, H’ is high in the western Mediterranean basin including the Tyrrhenian and Alboran Seas, the waters around Sardinia and the Strait of Sicily. However, there is an area of comparatively low diversity values in the eastern coast of the Iberian Peninsula around the Ebro river mouth. Regarding the eastern basin, the Adriatic and Southern Aegean Seas have moderate and high H’ values, whereas the waters around Crete showed the lowest values. Species richness was also high in the whole western basin, including the area around the Ebro mouth. In the eastern basin, S was high in the Ionian Sea and in the western part of the Southern Aegean; it was comparatively low in the northern and southern Adriatic Sea and around Crete. Overall, no consistent pattern in diversity and species richness were found along the Mediterranean.

**Fig 9:**
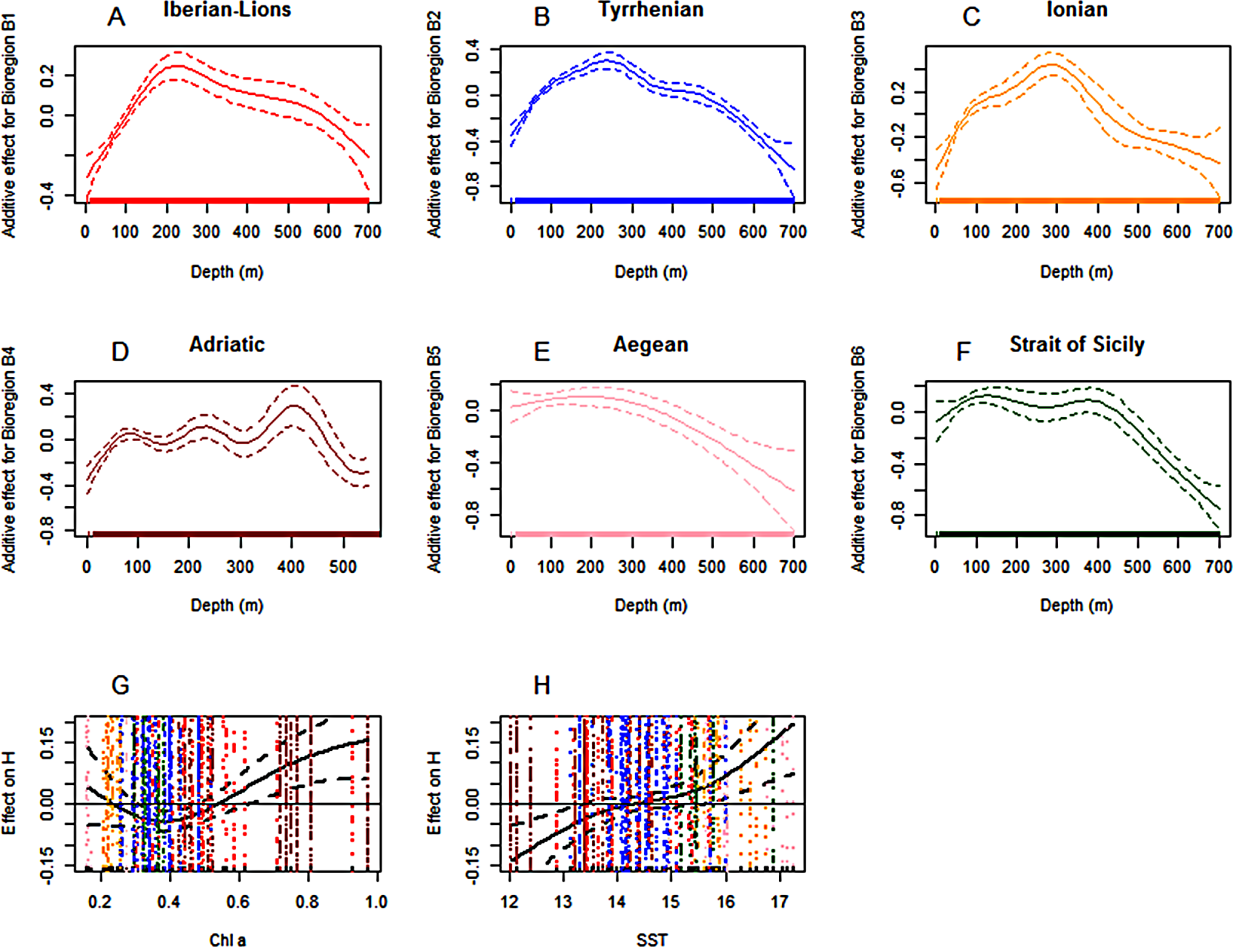
GAM outputs of partial effects for 1) depth at the six bioregions investigated (A-F), 2) chlorophyll concentration (Chla; G) and 3) surface sea temperature (SST; H) on Mediterranean cephalopod diversity H’. Solid lines indicate the fitted partial effects and broken lines the 95% confidence intervals (CI).

As the remaining variables analysed (depth, Chla and SST) displayed similar trends for H’ and S, only the results of the H’ model (Fig 9) are shown here (model outputs for S are given in S3 Fig). The smoothers for depth were humpback shaped in the Iberian-Lions, Tyrrhenian and Ionian Sea, with maxima at 200 in the two former regions and 300 m in the last one. In the Strait of Sicily and Aegean Sea, the effect decreased with depth but with a plateau at 100–400 m in the second case. The Adriatic Sea showed a completely different pattern, with a general increase with depth (punctuated with local maxima at about 100, 200 and 400 m depth) and a marked decrease in waters deeper than 400 m. In general, the strongest effect was found between 200 and 400 m depth, indicating that the highest diversity and species richness are likely to be found at the shelf break and upper slope.

The effect of Chla on diversity took an inverted bell shape with diversity declining with increasing Chla content until the inflection point at values close to 0.4 mg·m-3 (Fig 9G). After this point, diversity increased with increasing Chla. Partial residuals on the Chla effect indicates that the areas where Chla positively enhances diversity belong to the Southern Aegean Sea (pink dots) and the Adriatic (brown dots), which are the areas with the lowest and highest Chla respectively (Fig 9G).

SST showed a general positive effect on diversity, with below-average diversity values at low temperatures (<14–16°C) and higher values at higher temperatures (Fig 9H). The plotted partial residuals of the SST show that those below-average diversity values at high temperatures were mainly associated with the Strait of Sicily, the Ionian and the Aegean Sea (Fig 9H), while the negative part of the SST effect was partially associated with the Adriatic and the Iberian-Lions area.

## Discussion

The present study analysed spatio-temporal trends of cephalopod diversity in the whole Mediterranean Sea during the last two decades, using species richness and the Shannon-Wiener index as diversity descriptors. In spite of the intense sampling (18214 stations), the species accumulation curves did not level off in all areas, meaning that results should be carefully interpreted in a geographical context. Altogether 58 taxonomical units of cephalopods were found, of which 47 were determined to species level; these species account for 71% of the currently known Mediterranean cephalopod fauna [38].

### Spatio-temporal patterns

The spatial distribution of cephalopod diversity showed no clear longitudinal or latitudinal gradients at the whole basin scale. This result is independent from the scale considered (haul, area) or the diversity measurement used (H’, S), and differs from findings obtained analyzing unstandardized Mediterranean fish datasets [1,10,11,39,40]. Interestingly, the previous studies supporting a longitudinal trend of diversity are all based on data compiled from different data sources and cruises. On the other hand, a study on benthic Mediterranean deep-sea fauna combined different sampling programs with literature reviews and revealed that for most of the taxa analysed, a longitudinal gradient was only found regarding biomass and abundance, but not for diversity [41]. These authors concluded that longitudinal trends are apparently weak and inconsistent across different components of deep-sea biota. In agreement with this, none of the three studies which analysed standardized large-scale, long-time Mediterranean datasets, namely on groundfish diversity [12,15] and fish diversity in general [16], found a clear spatial gradient of diversity. Instead they suggested that some areas in the eastern basin were much more diverse than previously thought. Granger et al. [16] analyzed MEDITS data from 1994–2012 and found fish species richness per haul to be highest in the western areas of the western Mediterranean basin, in the eastern Ionian Sea, around Malta and in areas of the Aegean Sea. Gamma diversity, on the other hand, was lowest in the Adriatic but highest in the Balearic Sea, eastern Ionian Sea and the western part of the Southern Aegean Sea. Similar patterns were also found by Gaertner et al. [12,15] for groundfish communities on the Mediterranean shelf and slope. In a former study, D’Onghia et al. [42] already concluded that the North Aegean cephalopod diversity was as high as in any other Mediterranean area.

In spite of claims of biodiversity loss as a result of high anthropogenic impacts in the Mediterranean (fishing exploitation [31], pollution [32], climate change [29]), overall diversity of cephalopods has been rather stable over the last two decades. Such stability in species diversity with time also applies to fish [16]. Consistent temporal trends were not found at regional scales either, as cephalopod diversity oscillated annually in a saw-tooth shape without any clear tendency. The only striking feature is the high diversity values in the Southern Aegean Sea during recent years, which seems not to be related to Lesseptian migrants [43] as our data contain no records of Red Sea species. The lack of general trends in cephalopod and fish diversity during the last 20 years could be due to the fact that the ecosystem was already altered before the beginning of our time series or that noticeable changes will only be revealed at longer temporal scales.

### Drivers of diversity

The diversity of Mediterranean cephalopods followed a hump-shaped bathymetric pattern, being highest between about 200–400 m, which is in accordance with previous works on cephalopod assemblages in the area. However, additional bioregion-dependent bathymetric patterns were observed. In the central Mediterranean, species richness increased with depth up to a peak at about 200 m and then decreased down to 600 m depth [44]. In the western basin, both species richness and diversity were highest on deep shelf and upper slope grounds between 100–600 m compared to shallower (50–100 m) and deeper (600–800) waters [23]. A similar diversity pattern of decreasing species richness with depth at the whole Mediterranean scale was found for groundfish slope (>200 m) communities [15]. However, bathymetric patterns in this work varied according to the type of diversity index used, e.g. an opposite relationship was found between taxonomic distinctness and depth. In the California current, by contrast, the use of the distinctness index also revealed the hump-shaped pattern [45].

The observed diversity patterns could be related to the so-called mid-domain effect, which states that diversity in a bounded geographical domain is highest in the middle of that domain. The mid-domain peak is independent from environmental gradients and only due to the maximum overlap of species distribution ranges at that point [46]. However, when testing this hypothesis with fish data from different regions, Kendall and Haedrich [47] results did not tally with the mid-domain effect theory. Rosa et al. [7] came to the same conclusion in their study of large scale patterns in pelagic cephalopod diversity worldwide. Their study demonstrated highest diversity in the first 200 m but no evidence of the mid-domain effect. According to Levin et al. [48], the hump-shaped form might result from optimal conditions (e.g. productivity) near the shelf-break; these systems are highly productive and diverse habitats, often dominated by complex biocenoses [49]. Enhanced phytoplankton and zooplankton productions associated with shelf-break fronts have been observed in many ecosystems, and nurseries of numerous species are associated to shelf-break habitats in the Mediterranean [50–52]. Differences in the extension and morpho-geographical characteristics of shelf-break habitats might be responsible for the different bathymetric effects evidenced in the six bioregions investigated.

Productivity has often been proposed to influence diversity, and various authors investigated this relationship. Our results indicate that low and high Chla seems to enhance species diversity, as the GAM analysis showed a U-shaped effect with positive effect sizes associated with the Aegean and Ionian Sea on one hand and the Iberian-Lions and Adriatic regions (high Chla) on the other hand. This suggests that different mechanisms are in play in different areas, probably because the effect of productivity interacts with other factors and therefore depends on the ecosystem properties. The positive link between Chla content and diversity in productive areas could be due to the fact that sufficient food resources enable less competitive species to survive better than in resource-limited environments, resulting in a higher evenness of the community [7,8]. Furthermore, more productive systems support more species and trophic levels [53]. In fact, a positive relation between net primary production and pelagic cephalopod diversity was found analyzing large-scale patterns worldwide, though this relation was very weak in coastal ecosystems [7,8]. For bacteria, there is experimental evidence showing that seasonal fluctuations in availability of limiting resources can favor biological diversity by the coexistence of different ecotypes via frequency dependent competition [54]. Based on regression analyses, McClatchie et al. [55] found a relationship between demersal fish diversity and surface phytoplankton biomass, but they state that this effect may not be causal. A negative effect of Chla, as seen in areas of intermediate Chla content, could be explained by the rise of a few dominant species or changes in food quality (a different phytoplankton community composition having consequences on higher trophic levels) [53]. However, high diversity values observed in the less productive areas of the Mediterranean were likely related to different ecological processes. We argue that high diversity in rather oligotrophic and warm areas, such as the eastern Ionian and the Aegean Sea, may result from closer coupling of the cycles of primary and secondary producers and the low seasonal amplitude of these cycles [7]. These characteristics of oligotrophic regions favor diversity [54,56]. An additional explanation is that the contribution of different species of cephalopods to community diversity in the Eastern Mediterranean is likely different, with higher contribution of species less sensitive to primary production variability and higher sensitivity to other environmental variables, for example temperature (Fig 9H) or rainfall [57]. A recent studies in the Western Mediterranean shows that, on a given area, two cephalopods species display contrasting responses to a given environmental variable [58].

Temperature is another parameter considered to influence species diversity via different pathways like biochemical rates, mutation rates or lifestyles [7]. In contrast with a previous work carried out in the western Mediterranean [25], which did not find any relationship between temperature and cephalopod diversity, our analysis revealed a positive correlation between these parameters, the effect being stronger for S than for H’. A positive correlation with S was also found in a work of Ben Rais Lasram et al. [1] comparing fish diversity in the whole Mediterranean with the mid-domain effect hypothesis [1]. In the Barents Sea, Johannesen et al. [59] found contrasting results depending on the diversity index used, since the SST–diversity correlation was negative and positive using H’ and S, respectively. Changes in temperature may yield competitive advantages to certain species due to their higher temperature tolerances and/or mobility, which may lead to changes in species turnover and community composition [9]. Apparently, warmer SST leads to higher species number ([59], this study), while the evenness can be affected either positively (this study) or negatively [59]. Species dominance may be a context-dependent question related to the community composition and ecosystem characteristics.

Apart from the drivers analyzed in our study, there are other factors influencing species diversity. The geographical effect included in our models may capture some of these effects not explicitly included, such as the influence of the shelf area extension due to its importance as nursery areas. In previous studies, for instance, topographic features were found to affect fish [55] and cephalopod diversity [26]. Diversity patterns of different deep-sea taxa were found to vary according to the system (e.g. slope, canyon, sea mount), evidencing the importance of topographic and ecological features [41]. Other studies reported that diversity depends on climate and shelf area, and gradients seem to be set by historical, geological and climatic events, external forcing and oceanographic boundaries rather than by the physiological response of organisms to climate [8,60]. The phenology of primary producers and how biological processes are coupled to them (e.g. diurnal, ontogenetic and reproductive migrations) would also be related to the spatial and temporal patterns observed [42]. Furthermore, climate change and human induced perturbations such as fishing exploitation and habitat pollution can cause important impacts on marine biodiversity [13].

### Diversity and climate change

According to the fifth report of the Intergovernmental Panel on Climate Change (IPCC) about progression and consequences of the climate change, temperature and precipitation will modify present regional climates. The observations show a clear increase in the temperature of the Earth’s surface and the oceans. With the described and forecasted changes in temperature, salinity, pH, nutrients and oxygen, it is likely that Chl *a* concentration will be altered simultaneously. According to the report, oligotrophic provinces already expanded at average rates of 0.8 to 4.3% per year between 1998 and 2006, probably due to the reduction in nutrient availability owing to increased stratification of water masses. Given the relationship between cephalopod diversity, SST and productivity (Chla), ongoing climate change will probably lead to changing spatial and temporal diversity patterns of these species [61,62]. While expected raising temperatures might favour an increase in species richness according to our models, the effect of changed Chla concentration might depend on the specific bioregion. With the expected decrease of Chla concentration, diversity might decrease in less oligotrophy areas like the Adriatic Sea and the Iberian-Lions region (situated on the right part of the U-shaped Chla effect), while according to our model an increase in diversity would be possible in the Aegean Sea, the Ionia Sea and in some areas of the Strait of Sicily. However, according to different climatic models of a study of Macias et al. [63], these regions are more likely to experience an increase in Chla rather than a decrease, due to vertical density changes caused by a combination of warming and salinization. According to our model, diversity would therefore decrease in these areas as well.

## Conclusion

Our study, based on a standardized long-term Mediterranean-scale survey dataset, presents spatio-temporal patterns of cephalopod diversity measured by two different diversity indices. Cephalopod diversity showed no clear longitudinal or temporal trends over the last two decades. The spatio-temporal pattern of cephalopod diversity in the Mediterranean seems to be driven only partly by temperature and productivity gradients, as local physical and geographical factors like depth appear to be of influence as well. A useful expansion of the present study could be the integration of biotic factors (e.g. species aggregation, competition) as well as anthropogenic influences like pollution or fishing pressure [16]. Together with previous studies on fish diversity, the present results are of paramount importance to be used as a baseline scenario for future analyses of Mediterranean biodiversity over the next decades, such as the one foreseen by the European Marine Strategy Framework (Directive 2008/56/EC).

## Supporting Information

S1 Fig Map of the Mediterranean Sea showing the geographical sub-areas (GSAs) established by the General Fisheries Commission for the Mediterranean (GFCM) and the MEDITS stations sampled during 1994–2013 (20463 hauls). (TIF)

S2 Fig Species richness per GSA rarefied to 20 hauls. Samples included from 1994–2012.
(TIFF)

S3 Fig GAM outputs of partial effects for 1) depth at the six bioregions investigated (A-F), 2) chlorophyll a concentration (Chla; G) and 3) surface sea temperature (SST; H) on Mediterranean cephalopod richness S. Solid lines indicate the fitted partial effects and broken lines the 95% confidence intervals (CI).
(TIFF)

S1 Table List of all species found during MEDITS, by area. **Numbers are frequency of occurence averaged from 1994–2012.** Species marked in grey were excluded from the modeling. *Alloteuthis media*, *Alloteuthis subulata* and *Alloteuthis* sp. were joined for the analysis.
(PDF)

S2 Table Best model selection for H’ and S based on explained variance, GCV and AIC. All variables shown in the model formulations were significant. Best model marked in grey.
(PDF)
