# Supplementary figures and images for: Large-Scale Spatio-Temporal Patterns of Mediterranean Cephalopod Diversity
Source: PLoS One. 2016 Jan 13;11(1):e0146469. doi: 10.1371/journal.pone.0146469 (PMC4712019; doi:10.1371/journal.pone.0146469)

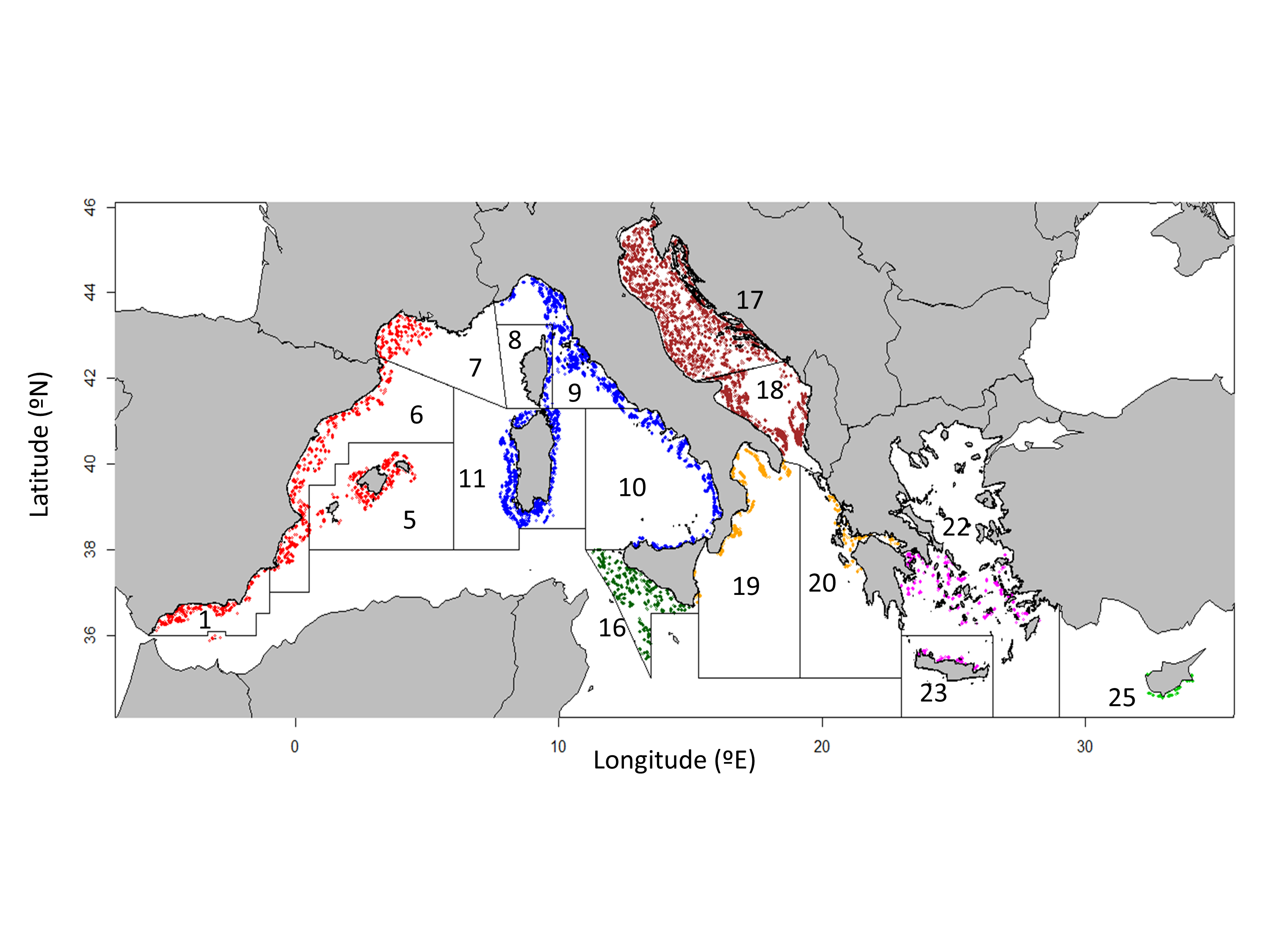

Supplement: S1 Fig — (TIF) [file pone.0146469.s001.tif]

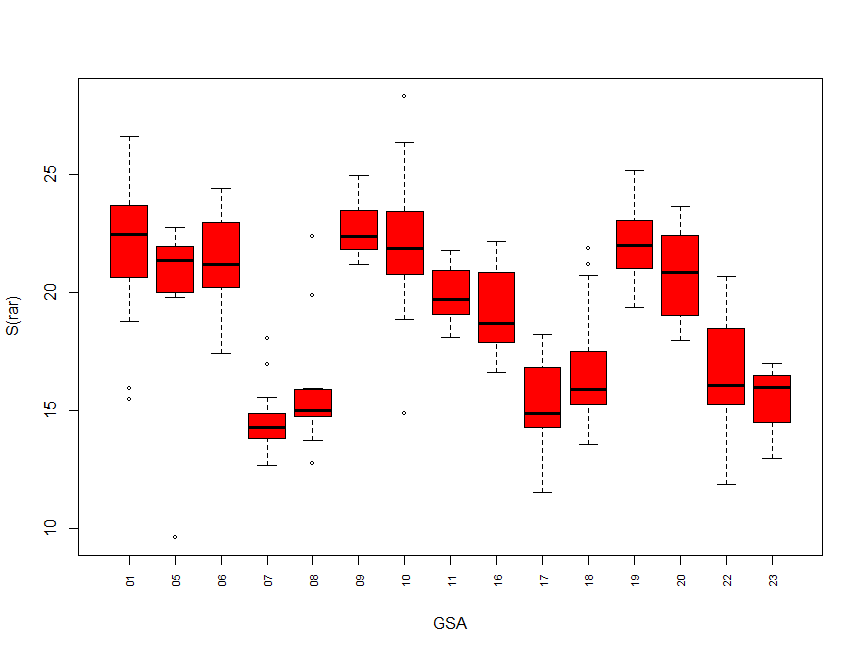

Supplement: S2 Fig — Samples included from 1994–2012. (TIFF) [file pone.0146469.s002.tiff]

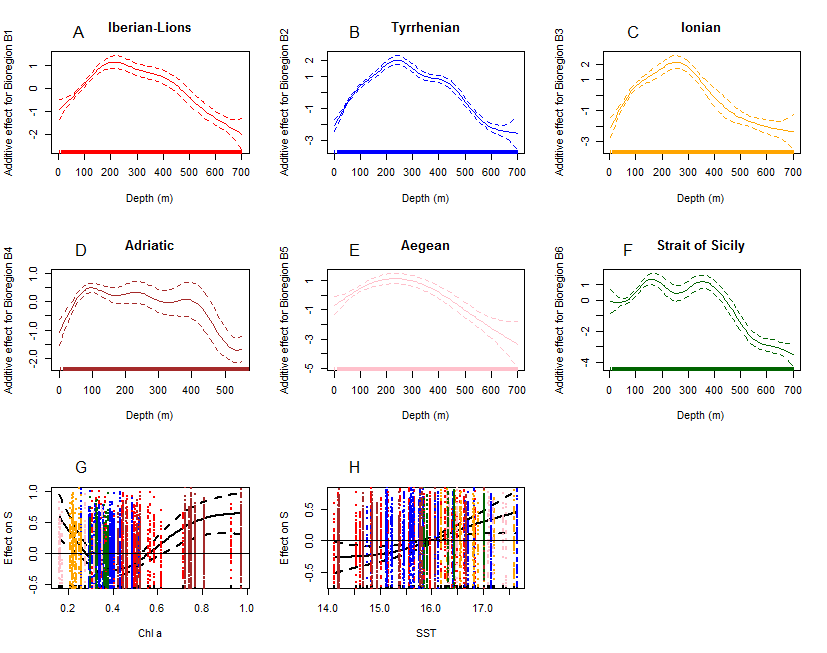

Supplement: S3 Fig — Solid lines indicate the fitted partial effects and broken lines the 95% confidence intervals (CI). (TIFF) [file pone.0146469.s003.tiff]
