# Supplementary material for: Large-Scale Spatio-Temporal Patterns of Mediterranean Cephalopod Diversity
Source: PLoS One. 2016 Jan 13;11(1):e0146469. doi: 10.1371/journal.pone.0146469 (PMC4712019; doi:10.1371/journal.pone.0146469)
Supplement: S1 Table — Numbers are frequency of occurence averaged from 1994–2012. Species marked in grey were excluded from the modeling. Alloteuthis media, Alloteuthis subulata and Alloteuthis sp. were joined for the analysis. (PDF) [file pone.0146469.s004.pdf]

Table S1: List of all species found during MEDITS, by area. Numbers are frequency of occurence averaged from 1994-2012. Species marked in grey were excluded from the modeling. *Alloteuthis media*, *Alloteuthis subulata* and *Alloteuthis* sp. were joined for the analysis.

Species	Iberian-Lions	Tyrrhenian	Ionian	Adriatic	Aegean	Strait of Sicily
**Order SEPIIDA**						
**Family Sepiidae**						
Sepia elegans Blainville, 1827	28.84	23.13	29.76	31.93	38.94	26.42
Sepia officinalis Linneus, 1758	6.79	6.54	3.76	9.17	15.83	9.50
Sepia orbignyana Férussac, 1826	20.69	28.40	14.08	14.58	55.28	20.01
Sepia spp.	0.23	0.56	0.00	0.35	0.00	2.16
**Family Sepiolidae**						
Sepiola affinis Naef, 1912	0.15	0.05	0.14	0.41	0.00	0.29
Sepiola intermedia Naef, 1912	0.75	0.43	4.04	1.23	0.25	2.45
Sepiola ligulata Naef, 1912	0.08	0.38	1.39	0.71	0.00	0.00
Sepiola robusta Naef, 1912	0.40	0.14	0.91	2.79	0.00	0.00
Sepiola rondeleti Leach, 1817	0.03	0.74	0.77	0.24	0.00	0.36
Sepiola spp.	17.05	9.35	2.37	18.67	5.53	14.33
Rondeletiola minor Naef, 1912	8.02	11.89	19.09	8.50	2.26	5.04
Sepietta obscura Naef, 1916	0.05	0.58	0.00	0.54	0.00	0.00
Sepietta neglecta Naef, 1916	0.20	0.37	0.07	1.21	0.00	0.00
Sepietta oweniana (D'Orbigny in Férussac & d'Orbigny)	20.87	29.16	21.46	9.54	3.02	17.85
Sepietta spp.	0.05	4.03	0.14	7.59	0.00	0.29
**Unid. Sepiolinae**	0.00	0.00	0.00	0.00	18.34	4.39

Rossia macrosoma (Delle Chiaje, 1830)	10.56	14.29	11.08	5.32	17.59	11.52
Neorossia caroli (Joubin, 1902)	3.87	7.40	5.09	1.77	2.01	4.18
Heteroteuthis dispar (Ruppell, 1844)	2.34	2.70	5.51	0.28	1.51	0.50
Stoloteuthis leucoptera (Verrill, 1878)	0.63	0.24	0.00	0.00	0.00	0.00
**ORDER MYOPSIDA**						
**Family Loliginidae**						
Alloteuthis media (Linnaeus, 1758)[1]	52.25	21.99	30.10	66.32	14.07	37.15
Alloteuthis subulata Lamarck, 1798[1]	27.58	3.87	2.44	7.83	0.25	11.45
Alloteuthis spp.[1]	3.60	10.61	0.00	0.02	27.64	2.16
Loligo forbesii Steenstrup, 1856	3.65	15.51	5.02	0.67	28.64	5.33
Loligo vulgaris Lamarck, 1798	13.08	16.58	15.05	36.97	24.62	17.64
Loligo spp.	1.63	0.66	0.00	0.00	1.51	0.00
**ORDER OEGOPSIDA**						
**Family Ommastrephidae**						
Ommastrephes bartramii (LeSueur, 1821)	0.00	0.00	0.00	0.00	0.00	0.07
Illex coindetii (Verany, 1839)	45.54	45.78	53.80	64.48	72.36	52.41
Todarodes sagittatus (Lamarck 1798)	21.75	14.16	12.20	4.82	16.58	28.01
Todaropsis eblanae (Ball, 1841)	12.70	35.12	27.87	20.35	5.28	43.12
**Family Histioteuthidae**						
Histioteuthis bonnellii (Férussac, 1835)	3.22	6.49	6.83	1.38	0.25	7.27
Histioteuthis reversa (Verrill, 1880)	4.40	6.36	14.36	2.25	1.01	2.66
Histioteuthis spp.[3]	0.10	0.00	0.07	0.02	0.00	0.94

**Family Onychoteuthidae**						
Ancistroteuthis lichtensteinii Orbigny, 1839	4.32	0.80	3.97	0.11	0.75	0.07
Onychoteuthis banksii (Leach, 1817)	0.50	0.11	0.63	0.09	0.50	0.29
**Family Enoploteuthidae**						
Abralia veranyi (Rüppell, 1844)	9.18	10.76	21.53	3.07	1.26	16.27
Abraliopsis morisii (Vérany, 1839)	0.00	0.00	0.07	0.00	0.00	0.00
Unid. Enoploteuthidae	0.00	0.00	0.00	0.00	0.75	0.00
**Family Chtenopterygidae**						
Chtenopteryx sicula (Veranyi, 1851)	0.00	0.03	0.07	0.00	0.00	0.00
**Family Octopoteuthidae**						
Octopoteuthis sicula Rüppell, 1844	0.00	0.00	0.42	0.00	0.25	0.00
**Family Chiroteuthidae**						
Chiroteuthis veranii (Férussac, 1835)	0.00	0.02	0.70	0.00	0.00	0.00
**Family Ancistrocheiridae**						
Ancistrocheirus lesueurii (Orbigny, 1842)	0.00	0.00	0.28	0.00	0.00	0.00
**Family Brachioteuthidae**						
Brachioteuthis riisei (Steenstrup, 1882)	0.78	0.00	0.35	0.00	0.75	0.00
**Family Pyroteuthidae**						
Pyroteuthis margaritifera (Rüppell, 1844)	0.00	0.00	0.21	0.00	0.00	0.00
**ORDER OCTOPODA**						
**Family Octopodidae**						
Octopus vulgaris Cuvier, 1798	34.60	21.63	16.52	9.39	29.40	16.70
Callistoctopus macropus Risso, 1826	0.25	0.42	0.28	0.48	0.00	0.36

Octopus salutii (Verany, 1839)	10.91	12.50	7.25	6.06	0.50	8.35
Macrotritopus defilippi (Vérany, 1851)	2.29	0.88	0.07	0.39	0.00	0.36
Octopus spp.	0.08	0.16	0.00	0.13	0.00	0.07
Pteroctopus tetracirrhus (Delle Chiaje, 1830)	6.21	16.08	4.74	1.08	2.76	10.37
Scaeurgus unicirrhus (Orbigny, 1840)	7.87	23.61	21.39	3.40	28.39	25.99
Bathypolypus sponsalis (P. & H. Fischer, 1892)	9.38	3.87	0.14	0.00	0.00	0.65
Eledone cirrhosa (Lamarck, 1798)	62.91	63.41	27.32	36.97	18.34	30.09
Eledone moschata (Lamarck, 1798)	18.83	13.91	11.92	33.85	33.17	24.91
**Family Ocythoidae**						
Ocythoe tuberculata Rafinesque, 1814	0.03	0.00	0.00	0.00	0.00	0.00
**Family Argonautidae**						
Argonauta argo Linnaeus, 1758	0.00	0.00	0.14	0.00	0.00	0.29
**Family Opisthoteuthidae**						
Opisthoteuthis calypso Villanueva et al., 2002	0.08	0.00	0.00	0.00	0.00	0.00
Opisthoteuthis spp.	0.03	0.00	0.00	0.00	0.00	0.00

[1] all three groups joined to "Alloteuthis"
