# Supplementary material for: Large-Scale Spatio-Temporal Patterns of Mediterranean Cephalopod Diversity
Source: PLoS One. 2016 Jan 13;11(1):e0146469. doi: 10.1371/journal.pone.0146469 (PMC4712019; doi:10.1371/journal.pone.0146469)
Supplement: S2 Table — All variables shown in the model formulations were significant. Best model marked in grey. (PDF) [file pone.0146469.s005.pdf]

Table S2: Best model selection for H’ and S based on explained variance, GCV and AIC. All variables shown in the model formulations were significant. Best model marked in bold.

No	Model	R2	GCV	k	AIC
1	H' ~ Year +Region +s(lat, long)+ depth	15.8	0.18087	-	7079
2	H' ~ Year +s(lat, long)+ depth * region	18.2	0.17627	-	6917
3	H' ~ Year +s(lat, long)+ depth * region + s(Chl Spring) + s(SST Spring)	18.3	0.17626	K=4/4	6916
4	H' ~ Year +s(lat, long)+ depth * region + s(Chl Spring) + s(SST Winter)	18.4	0.17583	K=4/4	6901
5	H' ~ Year +s(lat, long)+ depth * region + s(Chl Winter) + s(SST Spring)	18.5	0.17577	K=4/4	6899
**6**	**H' ~ Year +s(lat, long)+ depth * region + s(Chl Winter) + s(SST Winter)**	**18.5**	**0.17572**	**K=4/4**	**6897**

No	Model	R2	GCV	k	AIC
7	S ~ Year +Region + s(lat, long)+ depth + region	33.2	2.6188	-	23850
8	S ~ Year +s(lat, long)+ depth * region	35.5	2.5419	-	23662
9	S ~ Year +s(lat, long)+ depth * region + s(Chl Spring) + s(SST Spring)	35.6	2.5371	K=4 /4	23650
10	S ~ Year +s(lat, long)+ depth * region + s(Chl Spring) + s(SST Winter)	35.5	2.5417	k=4 /4	23662
**11**	**S ~ Year +s(lat, long)+ depth * region + s(Chl Winter) + s(SST Spring)**	**35.9**	**2.5275**	**K=4 /4**	**23627**
12	S ~ Year +s(lat, long)+ depth * region + s(Chl Winter) + s(SST Winter)	35.8	2.5313	k=4 /4	23636
